# Autotrophic Fixed-Film Systems Treating High Strength Ammonia Wastewater

**DOI:** 10.3389/fmicb.2020.551925

**Published:** 2020-09-08

**Authors:** Hussain Aqeel, Steven N. Liss

**Affiliations:** ^1^School of Environmental Studies, Queen’s University, Kingston, ON, Canada; ^2^Department of Chemistry and Biology, Ryerson University, Toronto, ON, Canada; ^3^Department of Microbiology, Stellenbosch University, Stellenbosch, South Africa

**Keywords:** biofilms, moving bed biofilm reactors, hybrid systems, BioCord^TM^, ammonia removal, microbial community dynamics, nitrifying and denitrifying bacteria

## Abstract

The aim of the study was enrichment of nitrifying bacteria and to investigate the potential of autotrophic fixed-film and hybrid bioreactors to treat high strength ammonia wastewater (up to 1,000 mg N/L). Two types of fixed-film systems [moving bed biofilm reactor (MBBR) and BioCord^TM^] in two different configurations [sequencing batch reactor (SBR) and a continuous stirred tank reactor (CSTR)] were operated for 306 days. The laboratory-scale bioreactors were seeded with activated sludge from a municipal wastewater treatment plant and fed synthetic wastewater with no organics. Strategies for acclimation included biomass reseeding (during bioreactor start-up), and gradual increase in the influent ammonia concentration [from 130 to 1,000 mg N/L (10% every 5 days)]. Stable ammonia removal was observed up to 750 mg N/L from 45 to 145 days in the MBBR SBR (94–100%) and CSTR (72–100%), and BioCord^TM^ SBR (96–100%) and CSTR (92–100%). Ammonia removal declined to 87% ± 6, in all bioreactors treating 1,000 mg N/L (on day 185). Following long-term operation at 1,000 mg N/L (on day 306), ammonia removal was 93–94% in both the MBBR SBR and BioCord^TM^ CSTR; whereas, ammonia removal was relatively lower in MBBR CSTR (20–35%) and BioCord^TM^ SBR (45–54%). Acclimation to increasing concentrations of ammonia led to the enrichment of nitrifying (*Nitrosomonas*, *Nitrospira*, and *Nitrobacter*) and denitrifying (*Comamonas*, *OLB8*, and *Rhodanobacter*) bacteria [16S rRNA gene sequencing (Illumina)] in all bioreactors. In the hybrid bioreactor, the nitrifying and denitrifying bacteria were relatively more abundant in flocs and biofilms, respectively. The presence of dead cells (in biofilms) suggests that in the absence of an organic substrate, endogenous decay is a likely contributor of nutrients for denitrifying bacteria. The nitrite accumulation and abundance of denitrifying bacteria indicate partial denitrification in fixed-film bioreactors operated under limited carbon conditions. Further studies are required to assess the contribution of organic material produced in autotrophic biofilms (by endogenous decay and soluble microbial products) to the overall treatment process. Furthermore, the possibility of sustaining autotrophic nitrogen in high strength waste-streams in the presence of organic substrates warrants further investigation.

## Introduction

Increasing populations, urbanization, and commercial activities contribute to high strength wastewaters rich in organics and/or ammonia. Landfill leachate, return sludge from the anaerobic digesters, and several industries (e.g., oil refining, fertilizer production and use, and meat processing) can generate high strength ammonia wastewaters ranging from 500 to 2,500 mg N/L (Savci, 2012; Syron et al., 2015; Tabassum et al., 2018). Nutrients entering water bodies poses a serious threat to aquatic life and human health (Constable et al., 2003). Effluent regulations require treatment options and new approaches to treating high strength wastewaters in order to ensure water resources are protected.

Nitrifying bacteria oxidize ammonia to nitrate in two steps: in the first step, ammonia-oxidizing bacteria (AOB) (e.g., *Nitrosomonas*) oxidize ammonia to nitrite, and in second step nitrite-oxidizing bacteria (NOB) (e.g., *Nitrospira*) oxidize nitrite to nitrate. However, a subgroup of *Nitrospira* (comammox) can oxidize ammonia directly to nitrate in one step (Daims et al., 2015). There are several factors that can influence nitrification, including free nitrous acid, and the development and accumulation of sufficient nitrifying biomass (Yao and Peng, 2017; Tabassum et al., 2018; Du et al., 2019). The relative abundance of nitrifying bacteria in biomass is typically low since nitrifying bacteria are relatively slow-growing microorganisms, and are sensitive to environmental and operational stresses (Yao and Peng, 2017; Tabassum et al., 2018). The proportion of nitrifying bacteria observed in the biological nutrient removal systems varies and has been reported to be as low as 0.4% in a full-scale activated sludge wastewater treatment plant (Dionisi et al., 2002), 8.7% in a nitrifying sequencing batch reactor (SBR) (Li et al., 2007), 13% in biofilms and 17% in suspended biomass of a hybrid bioreactor (You et al., 2003), and as high as 41% in moving bed biofilm reactors (MBBR) (Hoang et al., 2014).

The partial nitritation and anammox (PN/A) is an attractive energy-efficient technology for the treatment of high strength ammonia wastewater (Lackner et al., 2014; van Loosdrecht and Brdjanovic, 2014). The application of PN/A technology is dependent on balancing the competition between the ammonia oxidizing bacteria (AOB), nitrite oxidizing bacteria (NOB), and anammox bacteria. Several operational conditions (e.g., dissolved oxygen concentration and temperature) have been engineered to support the growth of anammox bacteria, limit AOB and inhibit NOB (Lackner et al., 2014; Aqeel et al., 2019). The NOB are selectively washed-out from the system by elevating the temperature (>30°C). Therefore, the PN/A technology is applied to treat the digester effluent, on the side-stream; whereas, the relatively low temperatures of the mainstream wastewater is a challenge for the application of PN/A technology (Lackner et al., 2014; Laureni et al., 2019). Alternatively, partial denitrification and anammox (PD/A) technology has the potential to treat mainstream high strength ammonia wastewater. The PD/A process is advantageous (compared to the PN/A) because it does not require the operational conditions to washout the NOB. In the PD/A process, nitrates are partially denitrified to nitrite that is used by the anammox bacteria resulting in nitrogen removal from the system (Du et al., 2019; Zhang et al., 2020).

Fixed-film technologies are attractive due to its small footprint, and capability to support the slow-growing microorganisms (Mahendran et al., 2012; Wang et al., 2019). The first full-scale installations of MBBR and bio-cord or bio-lace, were first reported in the mid-1990s (Rusten et al., 1995; Randall and Sen, 1996). MBBR technology is relatively more widely adopted worldwide for biological nutrient removal (Forrest et al., 2016). The biocord^TM^ is composed of a central cord that supports the rings of the interwoven cord. Several different polymers have been tested to optimize the performance of biocord^TM^ (Tian et al., 2019). Both the MBBR and biocord^TM^ based bioreactors can be easily set up within conventional wastewater treatment plants to increase the capacity and efficiency of biological nutrient removal.

We report on a study that investigated the potential of autotrophic nitrifying fixed-film bioreactors to treat high strength ammonia wastewater. The main objectives of this study included: (a) an investigation of the acclimation of biomass to high strength ammonia wastewater (up to 1,000 mg/L) and enrichment of the nitrifying bacteria, and (b) a comparative examination of distinctive hybrid and fixed-film bioreactors to treat high strength ammonia wastewater.

## Materials and Methods

### Experimental Setup

Four laboratory-scale fixed-film bioreactors with an effective working volume of 2.2 L each, were operated for 10 months. Two configurations of fixed-film systems, including moving bed biofilm reactor (MBBR) and BioCord^TM^, each set up as a sequencing batch reactor (SBR) and continuous stirred tank reactor (CSTR) configuration, were operated for 10 months. The MBBR SBR was a hybrid bioreactor because it has a relatively higher amount of mixed liquor suspended solids (MLSS) along with the fixed-films. The BioCord^TM^ (Bishop Water Technologies, Canada) carrier media is composed of interwoven rings of polymers (vinylon and polypropylene) attached to a central cord to support the growth of the attached biomass (Supplementary Figures S1, S2). The same BioCord^TM^ carrier media was used for the SBR and CSTR configuration. The MBBR carrier media were made of polyethylene. The total available surface area for the fixed-films in the MBBRs (protected surface area) and BioCord^TM^ bioreactors were maintained in the range of 0.58–0.62 m^2^. A range of surface area is given to reflect the loss of carrier media during sampling. The protected surface area of the MBBR carrier media was used for the comparison owing to little or no biofilm accumulating on the outer surface of the MBBR media (Mahendran et al., 2012).

All bioreactors were equipped with peristaltic pumps for feeding and draining (Cole Parmer model 7520-35), air pumps (Optima LR-91926), and pH meters. All bioreactors were fed and aerated (with diffused air store) from the bottom of the bioreactors. The CSTRs were drained from the top of the bioreactors at 2.2 L mark; whereas, the SBRs were drained from the draining port at 1.1 L mark on the bioreactors. The SBRs were programmed (using programmed timers) for the 12 h sequencing batch cycles (15 min of feeding, 11 h and 15 min of reacting, 15 min of settling, and 15 min of draining phases). The CSTRs and SBRs were programmed to maintain a constant hydraulic retention time (HRT) of 24 h. The feeding and draining tubes were regularly either flushed or replaced to minimize any impact on operations as a result of clogging due to fouling. The HRT of the bioreactors was calibrated biweekly to maintain a constant HRT.

The treated effluent water was filtered (using a 0.45 μ syringe filter) prior to the measurement of ammonia, nitrite, and nitrate concentrations using HACH assays (HACH, London, ON, Canada). Dissolved oxygen (DO) and pH were measured using a DO probe (Oakton) and an online pH measurement system, respectively. The sludge volume index (SVI), mixed liquor suspended solids (MLSS) and effluent suspended solids (ESS) were measured following standard protocols (Eaton et al., 2005). The bioreactors were operated at room temperature (22°C ± 1), pH 7.5 ± 0.3, and dissolved oxygen (DO) concentration was maintained at 6–7 mg/L (Tian et al., 2019). The operational conditions of the bioreactors are summarized in Table 1.

**TABLE 1:** Operational conditions of the fixed-film and hybrid bioreactors.

	MBBR SBR	BioCord^TM^ SBR	MBBR CSTR	BioCord^TM^ CSTR
Biofilm carrier material	Polyethylene	Polypropylene and vinylon	Polyethylene	Polypropylene and vinylon
Surface area	0.58–0.6 m^2^	0.58–0.6 m^2^	0.58–0.6 m^2^	0.58–0.6 m^2^
Attached biomass (g/m^2^)	1.41	5.36	1.02	3.14
Live/dead cells	1.4 ± 0.2	3.0 ± 0.7	2.6 ± 0.3	2.7 ± 0.4
HRT (hours)	24	24	24	24
pH	7.5 ± 0.3	7.5 ± 0.3	7.5 ± 0.3	7.5 ± 0.3
DO	6–7 mg/L	6–7 mg/L	6–7 mg/L	6–7 mg/L

The attached biomass on the carrier media was measured on day 45, as previously described (Mahendran et al., 2012). Briefly, the carrier media were dried at 100°C for 1 h. After weighing, the carrier media were washed twice in 100 ml of 0.1 M NaOH solution at 80°C for 30 min. The clean carrier media were washed in running deionized water and dried at 100°C for 1 h. The difference in weight of the dry carrier media before and after the cleaning was used to estimate the attached biomass on the carrier media.

### Bioreactor Seeding

The bioreactors were seeded with activated sludge from a municipal wastewater treatment plant (Cataraqui Bay wastewater treatment plant, Kingston, ON, Canada). The final concentration of biomass following inoculation was 2 g/L MLSS seed in all bioreactors. The reactors were operated without wasting for the first 48 h. To further support biomass retention and biofilm formation, following an initial seeding, all bioreactors were reseeded on days 5, 10, 15, and 20. The biomass for reseeding was taken from a separate batch reactor (without biomass wasting) established and operated (for 5–10 days) under conditions similar to the experimental bioreactors (e.g., synthetic feed, aeration, pH, and HRT). All bioreactors were treated similarly, and reseeded for uniformity in bioreactor operations. The amount of biomass required for reseeding was calculated based on the hybrid MBBR SBR, to a final concentration of 2 g/L MLSS.

### Synthetic Wastewater and Ammonia Feeding Regime

**FIGURE 1:**
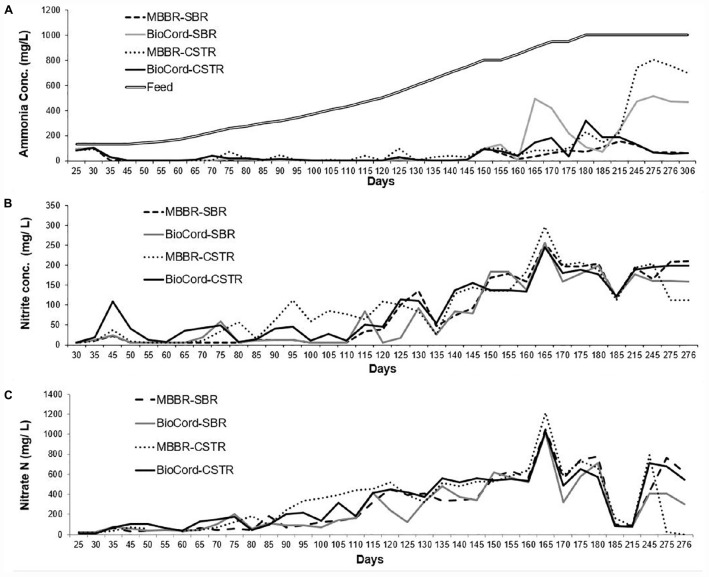
**(A)** Ammonia concentration in the synthetic feed and the final effluents of all bioreactors. **(B)** The nitrite accumulation in the final effluent, and **(C)** final nitrate effluent concentrations in al bioreactors.

The bioreactors were fed with ammonia-rich synthetic wastewater that was prepared (twice or thrice a week) with no organics. The recipe used in this study was a modified version of Tian et al. (2019). The macro and micronutrient composition for synthetic feed were (NH4)_2_SO_4_: 130–1,000 mg N/L, NaHCO_3_: 354.32 mg/L, MgSO_4_⋅7H_2_O: 70.98 mg/L, CaCl_2_⋅2H_2_O: 29.34 mg/L, KH_2_PO_4_: 79.09 mg/L, FeSO_4_⋅7H_2_O: 4.98 mg/L. The micronutrients were MnCl_2_⋅4H_2_O: 0.1 mg/L, Na_2_MoO_4_⋅2H_2_O: 0.025 mg/L, CuSO_4_⋅5H_2_O: 0.103 mg/L, CoCl_2_⋅6H_2_O: 0.001 mg/L, ZnSO_4_⋅7H_2_O: 0.03 mg/L. The startup ammonia concentration in the feed was 130 mg N/L. After an acclimatization period of 45 days, the ammonia concentration was increased by (approximately) 10% every 5 days, to a final ammonia concentration of 1,000 mg N/L in the synthetic feed on day 180 (Figure 1). However, when the ammonia removal efficiency declined below 90% (in all bioreactors), the influent ammonia concentration was kept constant for another period of 5 days. The ammonia removal declined below 90% twice on days (on days 150 and 165), and recovered to more than 90% within 10 days. The increase in the influent ammonia concentration was resumed to 10% every 5 days when ammonia removal efficiency recovered to higher than 90% in all bioreactors. The pH in all bioreactors was maintained at 7.5 ± 0.3 by adjusting the concentration of sodium bicarbonate in the synthetic feed. The feed tank was placed on the stirrer plate, and the synthetic feed was continuously mixed with a magnetic stirrer.

### Live/Dead Cells

Live/dead cell in the biofilms was estimated based on the fluorescent staining and confocal laser scanning microscopy (CLSM) (Zeiss LSM 710). The biofilm samples were collected on day 185 for the analysis. The MBBR and BioCord^TM^ carrier media were sliced with a surgical blade to obtain a cross-section of the biofilms. The samples were stained as described previously (Aqeel et al., 2016; Lishman et al., 2020). Briefly, a combination of SYTO 9 and propidium iodide stains were used for the estimation of live and dead cells, respectively. The biofilms were stained in a dark and humid Petri dish (2.5 cm diameter) for 30 min. After staining, samples were gently rinsed with deionized water to remove excess stain. All images were captured using a 10× magnification lens, using ZEN 2009 software (Zeiss LSM 710). ZEN 2012 (Zeiss) software was used for image analysis, including mean intensity measurements.

### DNA Extraction and Amplicon Sequencing

The biomass from the carrier media was collected as previously described (Mahendran et al., 2012; Lishman et al., 2020). Briefly, the biofilms were scrapped from the carrier media using a sterilized surgical blade and suspended in phosphate buffered saline. The suspension was centrifuged at 9,000 *g* for 10 min at 4°C, and the pellet was used for the genomic DNA extraction. Genomic DNA was extracted from the biofilms of all bioreactors and MLSS of MBBR SBR, using the PowerSoil DNA Isolation Kit (Mo Bio Laboratories Inc.). The compositions of the bacterial communities in biofilms and suspended biomass were assessed using 16S rRNA gene sequencing. The V3–V4 region of the 16S rRNA gene was amplified using primers 341F (CCTACGGGNGGCWGCAG) and 805R (GACTACHVGGGTATCTAATCC), with sample-specific barcodes. The PCR product was sequenced (Genome Quebec) using Illumina MiSeq. The forward and reverse sequences from the Illumina paired-end sequences were merged using the DADA2 software. The markers, adapters and the errors in the sequences (including chimera sequences) were removed using the DADA2 pipeline (Callahan et al., 2016), Taxonomic classification, α (based on observed species) and β (based on Bray–Curtis metrics) diversity were performed using Microbiome Analyst software. The assignment of the sequences was performed using SILVA taxonomy (Dhariwal et al., 2017; Chong et al., 2020). The low abundance (less than 10%) features were removed from the downstream analyses for α and β diversities. The graphs for the relative abundance of bacterial families and genera were prepared by selecting the top ten, and top 25 relatively abundant bacterial families, and by eliminating the bacterial genera with less than 35,000 sequences. A total of 5.9 million sequences were obtained that clustered into more than 13 thousand oligo taxonomic units. The predominant bacterial groups were selected to demonstrate the relative abundance of these microbes in the community. The less abundant bacterial genera or families are represented as “other” in the relative abundance graphs. The bioreactor sample metadata (accession number, SAMN14427038), and sequencing data (accession number, PRJNA614475) are available from the National Center for Biotechnology Information (NCBI) database^1^.

## Results

Fixed-film bioreactors (MBBR and BioCord^TM^) were operated in SBR and CSTR mode, where the MBBR SBR was a hybrid system consisting of a fixed-film and suspended growth. To support the acclimation of the biomass to increasing concentrations of ammonia, and enrichment of nitrifying bacteria, the influent ammonia concentration was gradually increased over the duration of the study from 130 to 1000 mg N/L at a rate of 10% every 5 days.

### Reactor Performance and Biomass Retention

Activated sludge from a municipal wastewater treatment plant was used to inoculate the bioreactors. Partial biomass washout from the laboratory-scale SBRs and CSTRs was anticipated due to the shift from municipal wastewater to synthetic wastewater, and relatively a short settling time of 15 min in the SBRs. A biomass reseeding strategy was employed for the development and retention of nitrifying bacteria through the early acclimation following start-up. The amount of biomass (for reseeding) in all bioreactors was calculated based on the hybrid MBBR SBR. The MLSS in the MBBR SBR was found to decline to 0.5–1.2 g/L MLSS (during first 20 days of bioreactor start-up). Bioreactors were supplemented with the biomass (acclimated to the autotrophic bioreactor conditions) to a final concentration of 2 g/L MLSS.

The MLSS in the hybrid bioreactor gradually increased from 400 ± 100 mg/L (on day 45) to 2,000 ± 600 mg/L by day 180 following startup and over the duration of increasing the influent ammonia concentration to 1,000 mg N/L. The MLSS gradually decreased in the BioCord^TM^ SBR and MBBR CSTR, and BioCord^TM^ CSTR to 15 ± 4, 37 ± 7, 25 ± 10, respectively, by day 180 (Table 2). The MLSS and effluent suspended solids (ESS) were the same in the fixed-film bioreactors. Overall, the ESS gradually declined in all bioreactors with time. The ESS was relatively lower in the BioCord^TM^ bioreactors compared to the MBBR bioreactors, for example, on days 120 and 180. In BioCord^TM^ reactors, the ESS was lower in SBR compared to the CSTR. Whereas in MBBRs, the ESS was higher in SBR compared to the CSTR (Table 2).

**TABLE 2:** Mixed liquor suspended solids (MLSS) and Effluent suspended solids (ESS) in the fixed-film and hybrid bioreactors.

	Ammonia conc. in feed (mg N/L)	MBBR SBR	BioCord^TM^ SBR	MBBR CSTR	BioCord^TM^ CSTR
MLSS (mg/L)	Seed	2000 ± 10	2000 ± 10	2000 ± 10	2000 ± 10
	130	400 ± 100	40–70	30–60	10–20
	250	1730 ± 50	20–70	30–60	34–140
	500	1720 ± 10	17–27	24–48	14–26
	1000	2000 ± 600	11–19	30–44	15–35
ESS (mg/L)	130	50–90	40–70	30–60	10–20
	250	20–90	20–70	30–60	34–140
	500	40–70	17–27	24–48	14–26
	1000	36–54	11–19	30–44	15–35

The SVI of the seed biomass was typical of a municipal activated sludge plant (154 ml/g MLSS), producing a good-quality effluent. It was observed that with a gradual increase in the influent ammonia concentration of synthetic wastewater, the SVI of the suspended biomass gradually decreased. The SVI of the suspended biomass in the hybrid system (MBBR SBR) was 123.0, 55.48, 33.8, and 27.69 at influent ammonia concentration of 130, 250, 500, and 1,000 mg N/L, respectively (Supplementary Figure S3).

The attached biomass on the carrier media was relatively higher in the MBBR and BioCord^TM^ SBRs compared to the respective CSTRs. The attached biomass on the MBBR SBR and CSTR were 1.41 and 1.01 g/m^2^, respectively, on day 45. The attached biomass on the BioCord^TM^ SBR and CSTR were 5.36 and 3.14 g/m^2^, respectively, on day 45 (Table 1). The developing biofilms on the BioCord^TM^ fibers were loosely bound and subject to detachment when fibers were sampled. The frequency of biofilm sampling was adjusted during biofilm development to minimize the destabilization of the reactor. The frequency of effluent sampling remained the same throughout the study. As the biofilm developed in the BioCord^TM^ bioreactors, there was minimal biomass detachment and low ESS during regular operation and sample collection from the bioreactors (Table 2).

**FIGURE 2:**
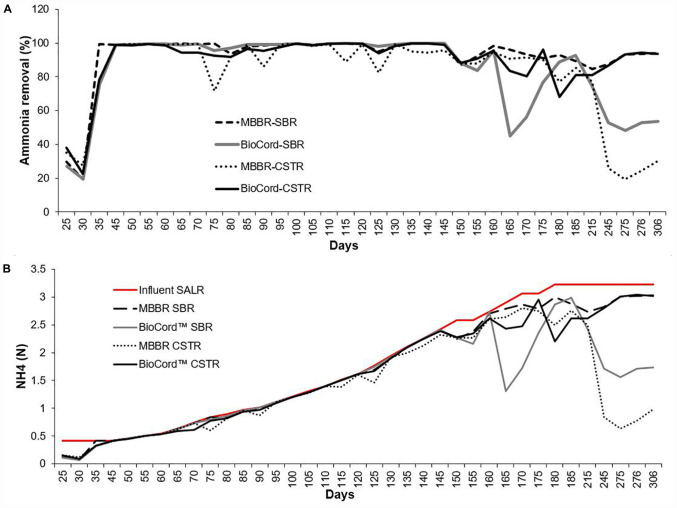
**(A)** The ammonia removal efficiency in the hybrid and fixed-film bioreactors. **(B)** The ammonia removal rate [NH_4_ (N) g/m^2^.day] in all bioreactors with the influent surface area loading rate (SALR).

The ammonia concentration (130 mg N/L) in synthetic feed was kept constant during the initial stabilization phase (45 days). The ammonia removal efficiency after seeding and reseeding increased from 30% ± 10 (on day 25) to more than 99% (by day 45), in all bioreactors (Figure 2). After an initial stabilization period of 45 days, the influent ammonia concentration was increased at the rate of about 10% every 5 days. The rate of increase in influent ammonia concentration was adjusted to 10% every 10 days (instead of every 5 days) when the ammonia removal efficiency decreased below 90%. This occurred on two occasions (at 800 and 900 mg N/L influent ammonia). The maximum influent ammonia concentration of 1,000 mg N/L was attained on day 180 and was maintained through day 300 of operation. Ammonia removal efficiency was relatively more stable in the MBBR SBR (94–100%), BioCord^TM^ SBR (96–100%), and BioCord^TM^ CSTR (92–100%) compared to the MBBR CSTR (72–100%) up to ammonia concentrations of 750 mg N/L, on day 145 (Figure 2). The ammonia removal efficiency at 1,000 mg N/L, on day 185, in both MBBR and BioCord^TM^ SBRs was 91% ± 2, and was relatively higher compared to the respective CSTRs (with an ammonia removal of 83% ± 2) (Figure 2). All bioreactors became unstable following day 215 of the bioreactor operation, with a decline in ammonia removal efficiency. The decline in ammonia removal efficiency may have been triggered by an operational stressor, unrelated to the high concentration of the influent ammonia. All bioreactors were capable of retaining the biomass during this phase, except the MBBR CSTR, where major biofilm sloughing and biomass washout was observed. Longer-term operation of the bioreactors at high strength ammonia concentrations (1,000 mg N/L) led to the recovery of elevated (93% ± 1), and stable ammonia removal efficiency in the MBBR SBR and BioCord^TM^ CSTR (days 275–306). However, ammonia removal in MBBR CSTR and BioCord^TM^ SBR declined to 20–30% and 48–54%, respectively, after long term operation of bioreactors (days 275–306) (Figure 2).

The surface area loading rate (SALR) was 0.42 NH^4^ (N) g/m^2^.day in all bioreactors, during the first 45 days following start-up. The SALR was gradually increased to 3.33 g N/m^2^.day, from 45 to 180 days (Figure 2). The surface area removal rate (SARR) was initially 0.09–0.15 NH_4_ (N) g/m^2^.day, and increased to 0.42 g N/m^2^.day on day 24. The ammonia removal rate was 0.42–2.39 g N/m^2^.day from 45 to 145 days. The SARR on day 185 was 2.9–3.0, and 2.6–2.8 g N m^2^.day in SBRs and CSTRs, respectively (Figure 2). The ammonia removal rate improved to 3.01–3.02 g N/m^2^.day in the hybrid MBBR SBR and BioCord^TM^ CSTR (from 275 to 306 days).

Overall, nitrate and nitrite accumulation was higher in the CSTRs compared to the SBRs. The concentrations of nitrite and nitrate were relatively lower in the BioCord^TM^ SBR and CSTR compared to the respective MBBR SBR and CSTR during the acclimation phase (Figure 1). There was a trend of gradual increase in the accumulation of nitrate and nitrite in the bioreactors with increasing influent ammonia concentrations. The total nitrate and nitrite accumulation on day 50 was 44 and 46 mg N/L in the MBBR and BioCord^TM^ SBR, respectively, indicating that 68–70% of nitrate and nitrite were removed from the system. The total nitrite and nitrate accumulation on day 110 [435 mg (NH_4_) N/L in feed] increased to 169 mg N/L in the MBBR and BioCord^TM^ SBR, corresponding to 61% of nitrate and nitrite being removed. The total nitrate and nitrite accumulation on day 180 [1,000 mg (NH_4_) N/L] was 980 and 914 mg N/L in the MBBR and BioCord^TM^ SBRs, respectively. After long term operation of the bioreactors (days 275–300), the nitrate and nitrite levels were relatively lower in the BioCord^TM^ SBR and MBBR CSTR compared to the MBBR SBR and BioCord^TM^ CSTR. The lower levels of nitrite and nitrate were observed in these bioreactors and correlated to the lower ammonia removal in the BioCord^TM^ SBR and MBBR CSTR (on day 306).

### Microbial Community Dynamics

**FIGURE 3:**
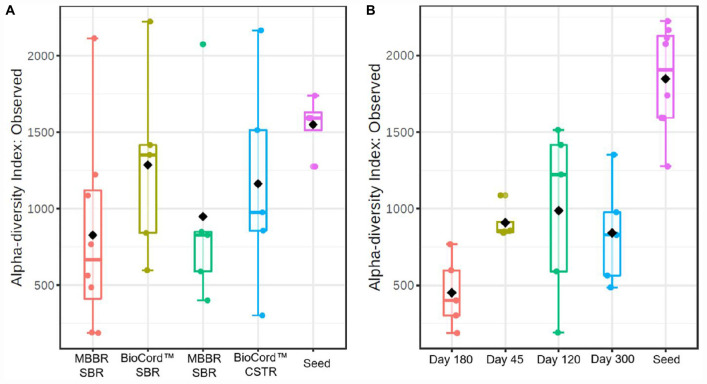
The box plots showing alpha diversity in hybrid and fixed film bioreactors **(A)**, and during the operation of the bioreactors at different time points **(B)**.

The rarefaction curves showing the alpha diversity of the bacterial communities indicate that the sequencing depth was enough to identify the rare microbial species. The microbial communities of the suspended biomass and biofilms were merged to represent the alpha diversity of the hybrid MBBR SBR. The alpha diversity index based on the observed species of the microbial communities shows that fixed-films of BioCord^TM^ reactors were more diverse compared to the MBBRs (Figure 3). Furthermore, the microbial communities of MBBR SBR and BioCord^TM^ SBR were relatively more diverse compared to the microbial communities of MBBR CSTR, and BioCord^TM^ CSTR, respectively. The alpha diversity of the samples collected at different time points shows that the group dispersion was relatively low in MBBR CSTR compared to the other bioreactors (Figure 3).

The alpha diversity of the seed biomass was higher and declined over the duration of the operation of the bioreactors (Figure 3). The observed species richness was lowest on day 180 (1,000 mg N/L influent ammonia). The microbial community diversity increased when the influent ammonia concentration was kept constant at 1,000 mg N/L for a relatively longer time period (from days 180 to 300). The group dispersion in the microbial communities of the bioreactors indicates that the species diversity in all bioreactors was relatively close to each other on day 45 (prior to increasing the concentration of ammonia in the feed); whereas, by day 120, and at an ammonia concentration of 500 mg N/L, the reactor communities (all bioreactors) were distinctive and diverse with respect to each other.

**FIGURE 4:**
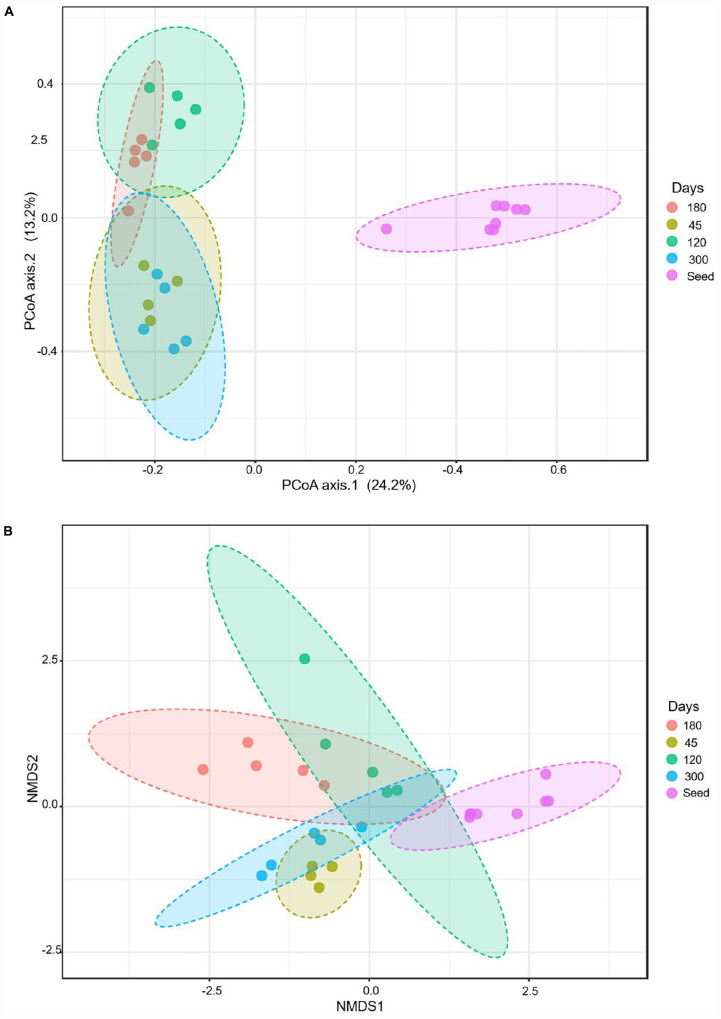
Beta diversity during evolutions of the microbial communities at different time points, based on Bray–Curtis metrics: **(A)** using principal coordinate analyses (PCoA), and **(B)** non-metric multidimensional scaling (NMDS).

The beta diversity index was characterized, based on the Bray–Curtis, using principal coordinate analyses (PCoA) and non-metric multidimensional scaling (NMDS). The PCoA measures fixed distances between the microbes based on the presence and absence of the bacterial species, genera or the families, whereas the NMDS also accounts for the abundance of a bacterial group. The PCoA analyses show that the seed biomass was very distinct from the microbial communities of the bioreactors over time. The microbial community continued to evolve during the operation of the bioreactors. The microbial communities of the seed biomass, on days 45 and 120 were distinct from each other; whereas, the microbial communities on day 180 overlapped the microbial communities of the bioreactors on days 120 and 300 (Figure 4). The NMDS analyses, accounting for the abundance of the bacterial groups, show that each of the reactor communities were distinct.

### Composition and Dynamics of Bacterial Populations in the Fixed-Films Communities

**FIGURE 5:**
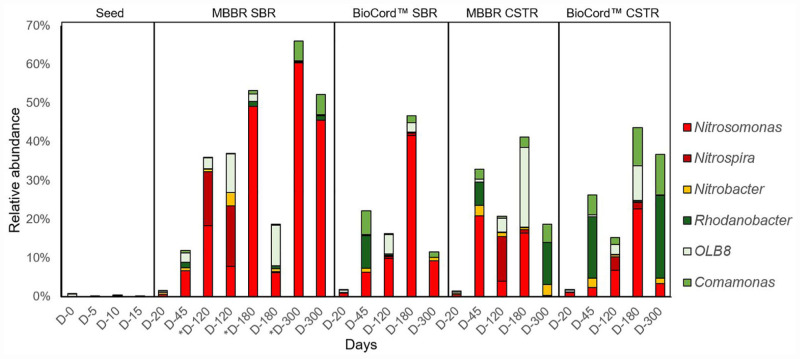
The relative abundance of predominant nitrifying and denitrifying bacterial genera in the hybrid and fixed-film bioreactors.

A total of 5,877,269 sequences from Illumina MiSeq were obtained. There was an average of 217,677 sequences per sample, which clustered into 13,288 oligo taxonomic units (OTUs). The OTUs were annotated based on SILVA taxonomy, where 5,546 OTUs were with two or more than two counts. A total of 1,138 low abundance features were removed based on prevalence (less than 10%). The number of features remaining after the data filtering steps was 4,408. The identification and abundance of microbial populations at the bacterial class and family level show that the fixed-film communities were very diverse (Supplementary Figures S4, S5). The microbial community analyses at the genus level using the top 10 predominant genera show that the nitrifying and denitrifying bacterial populations were predominant in fixed-film communities (Supplementary Figure S6). The predominant nitrifying and denitrifying bacterial populations were selected to understand the composition and dynamics of these bacteria in the communities of the hybrid and fixed-film biomass, treating high strength ammonia wastewater (Figure 5 and Supplementary Figure S7). It is important to note that estimations of the microbial communities were performed using amplicon sequencing, a semi-quantitative method revealing the relative abundance of the bacterial population in a microbial community.

#### Nitrifying Bacteria

The relative abundance of *Nitrosomonas*, in the microbial communities of all bioreactors, increased from the point of inoculation (0.03%), during reseeding (0.2–2.0%, day 20), stabilization (3–21%, by day 45) and acclimation phases, and gradually increased following increasing concentrations of influent ammonia from days 45 to 180 (Figure 5 and Supplementary Figure S7).

The relative abundance of *Nitrosomonas* was higher in the flocs compared to the biofilms of the hybrid bioreactor, and gradually increased from 6.3 to 18.4%, 49.2, and 60.4% on days 45, 120, 180, and 300 of operation, respectively. *Nitrosomonas* remained relatively constant (6–10%) in the biofilm community from days 45 to 180; however, the relative abundance in the biofilms of hybrid bioreactor increased more than sevenfold from days 180 to 300 (from 6.2 to 45.6%) at influent ammonia concentrations of 1,000 mg N/L. While the relative abundance of *Nitrosomonas* increased in the microbial communities of the flocs and biofilms in the hybrid bioreactor from days 180 to 300, a decrease was observed in the fixed-film bioreactors [BioCord^TM^ (SBR and CSTR) and MBBR CSTR] from days 180 to 300 (Figure 5 and Supplementary Figure S7).

The relative abundance of *Nitrosomonas* was highest (21% on day 45 of bioreactor operation) in the biofilms of MBBR CSTR compared to the other three bioreactor configurations (Figure 5). The MBBR CSTR biofilm was observed to be thinner with less biomass, compared to the other three configurations of the bioreactors, suggesting that *Nitrosomonas* was among the early biofilm colonizers that resulted in the highest relative abundance on carriers with relatively lower biomass. Levels of *Nitrosomonas*, *however*, were found to fluctuate in the MBBR CSTR throughout the duration of the study, increasing to 21% by day 45, followed by a decline to 4% by day 120, and then an increase to 16.4% by day 180. By day 300, the relative abundance of *Nitrosomonas* declined to 0.4% (Figure 5 and Supplementary Figure S7), and was associated with a major biofilm sloughing event and biomass washout from the MBBR CSTR as noted above (see section “Microbial Community Dynamics” acclimation of the bioreactors to high strength ammonia feed).

In the BioCord^TM^ systems, the relative abundance of *Nitrosomonas* bacteria was higher in the SBR compared to the CSTR configuration. The relative abundance of the *Nitrosomonas* in the fixed-films of the SBR was 6.3% by day 45 and increased to 9.9% by day 120 when the influent ammonia concentration was increased from 130 to 500 mg N/L. However, a greater than fourfold increase in the relative abundance of *Nitrosomonas* (41.7%) was observed by day 180 when influent ammonia concentration was increased from 500 to 1,000 mg N/L (Figure 5 and Supplementary Figure S7). The relative abundance of *Nitrosomonas* decreased to 9.4% by day 300 upon the end of the experimental period.

Within the BioCord^TM^ CSTR on day 45, the relative abundance of *Nitrosomonas* was estimated to be 2.4% (Figure 5). This increased approximately threefold (6.9%) from days 45 to 120 and more than threefold (22.8%) from days 120 to 180 when the influent ammonia concentration increased from 130 to 1,000 mg N/L by day 180. The relative abundance of *Nitrosomonas* declined to 3.5% by day 300; the same trend was observed in all fixed-film bioreactors where the relative abundance of *Nitrosomonas* declined when the influent ammonia concentration was kept constant at 1,000 mg N/L, from days 180 to 300.

*Nitrobacter* was estimated to be more abundant in the microbial communities of the MBBR CSTR and BioCord^TM^ CSTR (2.6 and 2.5%, respectively) compared to the flocs and biofilms of the hybrid bioreactor (0.8%) and biofilms of the BioCord^TM^ SBR (1.1%), on day 45. The relative abundance of *Nitrobacter* gradually declined in the microbial communities of all bioreactors (except in the biofilms of hybrid bioreactors) during the increase in the influent ammonia concentration. *Nitrobacter* increased to 3.4% in the microbial communities of the biofilms of the hybrid biofilms by day 120, followed by a decline to 0.7% by day 180. The relative abundance of *Nitrobacter* increased in the fixed-film communities of all bioreactors (except in the biofilms of the hybrid bioreactors), from days 180 to 300 when the influent ammonia concentration was kept constant at 1,000 mg N/L (Figure 5 and Supplementary Figure S6).

*Nitrospira* was relatively higher in the MBBR SBR (hybrid) and MBBR CSTR compared to the respective BioCord^TM^ SBR and CSTR (Figure 5 and Supplementary Figure S6). The relative abundance of *Nitrospira* spiked in the fixed-film (15.6%) and flocs (14.0%) of the hybrid bioreactor and the fixed-film (11.5%) of MBBR CSTR by day 120. Furthermore, in MBBR CSTR and hybrid bioreactors, the relative abundance *Nitrospira* was higher compared to the relative abundance of *Nitrosomonas* in the respective biofilm communities by day 120. In the BioCord^TM^ bioreactors, the abundance of *Nitrospira* was relatively higher in the microbial communities of CSTR biofilms (3.5%) compared to the SBR biofilms (0.6%) by day 120 when the influent ammonia concentration was at 500 mg N/L.

#### Denitrifying Bacteria

*Rhodanobacter*, *OLB8*, and *Comamonas* were the predominant denitrifying bacteria in the microbial communities of the hybrid and fixed-film bioreactors. The relative abundance of *Comamonas* was relatively higher in the BioCord^TM^ SBR (6.2%) and CSTR (5.1%) bioreactors compared to the MBBRs SBR (0.6%) and CSTR (2.6%) biofilms by day 45. The relative abundance of *Comamonas* declined to 0.2–2.0% in all bioreactors with the gradual increase in the influent ammonia concentration from 130 to 500 mg N/L, day 120. However, after an initial drop, the relative abundance of the *Comamonas* increased when the influent ammonia concentration was increased from 500 to 1,000 mg N/L. The relative abundance of *Comamonas* by day 180 was highest in the BioCord^TM^ CSTR (10.5%) followed by MBBR CSTR (2.7%), BioCord^TM^ SBR (1.7%), and MBBR SBR (0.3%) biofilms (Figure 5).

The relative abundance of *Rhodanobacter* followed a similar trend to *Comamonas*. *Rhodanobacter* was relatively higher in the biofilms of BioCord^TM^ SBR (8.5%) and CSTR (15.9%) compared to the MBBR SBR (1.3%) and CSTR (6%) by day 45. The relative abundance of *Rhodanobacter* was relatively higher by day 45 and by day 300 when ammonia influent concentrations were constant for extended periods of time compared to the periods leading to 120 and 180 when influent ammonia concentrations were gradually being increased. The abundance of *Rhodanobacter* was relatively higher in the biofilms of CSTRs BioCord^TM^ (21.4%) and MBBR (10.9%) compared to the respective SBRs by day 300 (Figure 5 and Supplementary Figure S7).

The relative abundance of *OLB8* throughout the experiment displayed a different trend compared to the other denitrifying bacteria (*Rhodanobacter* and *Comamonas*). The relative abundance of *OLB8* increased three to more than 10-fold during the phase when the influent ammonia concentration was gradually increasing (from days 45 to 180), followed by a decline in the relative abundance of *OLB8* when the influent ammonia concentration was kept constant from days 180 to 300. The relative abundance of *OLB8* was relatively higher in the biofilms of the hybrid bioreactor (2.5%) compared to the biofilms of fixed-film bioreactors (less than 0.1%), by day 45 (Figure 5). The relative abundance of *OLB8* was more than fourfold higher in biofilms (9.9 and 10.5% by days 120 and 180, respectively) compared to the flocs (2.8 and 2% by days 120 and 180, respectively) of the hybrid bioreactor (Figure 5 and Supplementary Figure S7). The relative abundance of *OLB8* increased (from less than 0.1%, on day 45) to 3.6 and 20.6% by days 120 and 180, respectively (in MBBR CSTR), and 2.6 and 8.9% by days 120 and 180, respectively (in BioCord^TM^ CSTR). Whereas, in the BioCord^TM^ SBR, the relative abundance of BioCord^TM^ increased to 5% by day 120, followed by a decline in relative abundance to 2.5% by day 180 (Figure 5 and Supplementary Figure S7).

The presence and abundance of heterotrophic denitrifying bacteria indicate that endogenous decay may have provided a source of organic substrate in the absence of organics in the influent wastewater. The presence of decaying cells in all biofilms was evident in microscopic images and image analyses. The ratio of live and dead cells in the biofilms was estimated using the fluorescent staining coupled with the confocal laser scanning microscopy. The live to dead ratio in the MBBR (SBR and CSTR), and BioCord^TM^ (SBR, and CSTR) was 1.4 ± 0.2, 2.6 ± 0.3, 3.0 ± 0.7, and 2.7 ± 0.4, respectively (Table 1).

## Discussion

This study provides unique insights into the development of the biofilms and changes in the microbial community of autotrophic nitrifying fixed-films and hybrid systems capable of treating high strength ammonia containing wastewaters. Two distinct fixed-film systems, MBBR and BioCord^TM^ in both SBR and CSTR configurations, were examined. The MBBR SBR represented a hybrid system consisting of both fixed and suspended growth forms. In anticipation of challenges in retaining biomass and growth of slow-growing autotrophs following inoculation from municipal activated sludge, a strategy was employed, including a reseeding regime from biomass adapted to initial conditions in the experimental reactors. The study demonstrates the potential for seamless acclimation to increasing concentrations of ammonia from 130 to 1,000 mg N/L over a period of 145 days. This period of acclimation was associated with the growth of nitrifying bacteria (*Nitrosomonas*, *Nitrospira*, and *Nitrobacter*) and denitrifying bacteria (*Comamonas*, *Rhodanobacter*, and *OLB8*). The relative distribution of the nitrifiers and denitrifiers in suspended biomass and fixed-films in the hybrid reactors, and the relative ammonia removal performance of the MBBR SBR and CSTR systems, suggests there are benefits to a hybrid system. The presence and abundance of the denitrifying bacteria in the bioreactors (fed without an organic substrate) indicates that endogenous decay and soluble microbial products, likely contribute to the growth of heterotrophic bacteria.

### Acclimation to High Strength Ammonia Wastewater

Reseeding is a common practice in the activated sludge system, where the settled biomass (in a separate tank) is reseeded in the system to maintain the higher biomass concentration for biological nutrient removal (Pehrson et al., 2008). The initial startup of the bioreactors entailed inoculating with the seed biomass, and a regime of reseeding to achieve a state where biomass levels were maintained over the initial 20 days to support the growth of slow-growing autotrophs in the biofilms. This was followed by a period of increasing concentrations of influent ammonia beginning on day 45. The biomass used for reseeding was developed from the original seed biomass and acclimated (in a batch bioreactor fed with a synthetic feed rich in ammonia lacking organic substrate) for 5–10 days before reseeding the experimental bioreactors. The selection and enrichment of autotrophs are essential because nitrifying bacteria are slow-growing autotrophs that require long solid retention time (Liu et al., 2004; Chung et al., 2007). Several studies have used biofilms or suspended biomass from nitrifying bioreactors to inoculate the bioreactors for easy startup and selection of the microbial community to treat high strength ammonia wastewater (Tian et al., 2019; Wang et al., 2019). The acclimation of the initial seed biomass was crucial because, in this study, the seed biomass was collected from the aeration tank of a municipal wastewater treatment plant. The acclimation of seed biomass resulted in gradual enrichment of *Nitrosomonas* from 0.03% (in seed biomass), to 0.2–2.0% (after reseeding on day 20), and 3–21% (by day 45), in all bioreactors.

Recently, the gradual increase in the influent ammonia concentration (from 54 to 800 mg N/L in 200 days) have been used for the optimization of partial denitrification/anammox (PD/A) technology (Du et al., 2019). In this study, the influent ammonia concentration was gradually increased (after 45 days of bioreactor startup) at a rate of approximately 10% every 5 days. The timing of the increase to influent ammonia was increased to 10 days when the ammonia removal efficiency decreased below 90%. This occurred on two occasions (at 800 and 900 mg N/L influent ammonia). The 10 days were sufficient for the recovery of ammonia removal efficiency. The goal was a seamless acclimation to support the enrichment of nitrifying bacteria to effectively treat high strength ammonia wastewater. The maximum relative abundance of nitrifying bacteria was observed in the hybrid bioreactor (60.4% in suspended biomass and 45.6% in fixed-films) on day 300. The relative abundance of nitrifying bacteria was 49.2%, 6.3% (in suspended biomass and fixed-films, respectively), 41.7, 16.9, and 24.4% in hybrid, BioCord^TM^ SBR, MBBR CSTR, and BioCord^TM^ CSTR, respectively, at the end of the gradual increase in the influent ammonia concentration on day 180 (Figure 5).

The gradual increase in the ammonia influent (up to 750 mg N/L, or 2.39 g N/m^2^.day) resulted in elevated ammonia removal (>98%) that was relatively more stable in the MBBR and BioCord^TM^ SBRs compared to the respective CSTRs (Figure 2). The ammonia removal efficiency declined in the SBRs (91% ± 2), and CSTRs (83% ± 2), when influent ammonia concentration was increased to 1,000 mg N/L. The maximum ammonia removal rate of 3 g N/m^2^.d was observed in the hybrid MBBR SBR and BioCord^TM^ CSTR (Figure 2). Tian et al. (2019) successfully employed BioCord^TM^ CSTR to treat a relatively high strength synthetic wastewater, but at lower concentrations than examined in this study (128 mg N/L), with ammonia removal efficiencies between of 92 and 97%. Several other studies (using different biofilm bioreactors) have shown relatively poor ammonia removal efficiency to treat high strength ammonia wastewater. For example, the ammonia removal efficiency was 75% in a hybrid membrane bioreactor (Downing and Nerenberg, 2007), and 87 and 38% in membrane-aerated biofilm bioreactors (Terada et al., 2003; Wang et al., 2019).

### Effectiveness of MBBRs and BioCord^TM^ in Treating High Strength Ammonia Wastewaters

The loss of planktonic and sloughed biomass can impede the startup of fixed-film and hybrid bioreactors. Growing and developing biofilm entails the involvement of bacteria cycling between fixed and planktonic forms (Bester et al., 2013). Developed films exhibit reversible and irreversible attachment, followed by sloughing (Telgmann et al., 2004; Aqeel et al., 2019). The sloughed biomass may be retained in the hybrid bioreactor and associate with the suspended biomass contributing to stable and elevated biological nutrient removal in the hybrid bioreactors. This was not the case in the MBBR CSTR where the sloughed biomass was washed out from the system. A decline in ammonia removal efficiency was observed in both MBBRs on day 215 (Figure 2). The ammonia removal efficiency improved to 93% ± 1 in the hybrid bioreactor, whereas a major biomass sloughing event and biomass washout in the MBBR CSTR resulted in poor ammonia removal efficiency (25% ± 10) that did not recover (days 245–306). Conditions that support higher biomass retention in a system can improve biological nutrient removal. Fixed-film, and hybrid systems, in particular, are more resilient to operational or nutritional stressors compared to conventional biological treatment systems (Mahendran et al., 2012; Aqeel et al., 2019).

Further insights into the stability of the reactors can be drawn from the alpha diversity analyses (observed species richness) based on group dispersion, which revealed that the microbial communities of the MBBR SBR were more diverse than the CSTR configuration (Figure 3). A biological nutrient removal system with a more diverse microbial community is likely more resilient and resistant to operational and nutritional stresses (Zhu et al., 2013; Aqeel et al., 2019).

The suspended biomass decreased initially (up to day 45), followed by a gradual increase in the hybrid bioreactor (Table 2). Biomass did decrease initially due to loss of slow settling biomass and selection of the fast settling biomass. The SVI of the suspended biomass decreased gradually with the time of operation of the bioreactors. The decrease in SVI of the suspended biomass was associated with an increase in the influent ammonia concentration and enrichment of the nitrifying bacteria and other changes in the community structure, leading to improved settling properties (Supplementary Figure S3). There was no evidence of granule formation or granular structures.

The structure of the BioCord^TM^ carrier media contributes to the entrapment of suspended biomass within the web of interwoven rings. The retention of loosely bound biomass resulted in higher biomass in the BioCord^TM^ reactors compared to the MBBRs. Early in the development of the biofilm, biomass loss during sampling was problematic due to loosely bound biomass that was readily detached. Over time a compact biofilm was established. The greater levels of attached biomass on BioCord^TM^ could be attributed to the ability to entrap biomass as well as the nature of aeration and hydrodynamic conditions compared to the MBBR system. In contrast, the MBBR media are subjected to physical interactions and hydrodynamic conditions that minimize biofilm accumulation, particularly on the outer surfaces of the carrier media, reducing the biomass accumulation in the MBBRs. Therefore, only the inner surface area of the MBBR media was used to standardize the surface area (Mahendran et al., 2012).

The attached biomass was relatively higher in the BioCord^TM^ SBR compared to the BioCord^TM^ CSTR (Table 2). It is suggested that the attached biomass was higher in the BioCord^TM^ SBR because the long SBR cycle (12 h cycles) allows the sloughed biomass to reattach to the carrier media. Whereas, in a BioCord^TM^ CSTR, the sloughed biomass was more prone to be washed-out from the system as indicated by the consistently higher ESS from the BioCord^TM^ CSTR compared to the SBR (Table 2). Biomass loss coincided with the decline in ammonia removal. The higher biomass retention in the BioCord^TM^ SBR resulted in relatively elevated and consistent ammonia removal during the stable phase (from 45 to 145 days).

### Dynamics of Nitrifying and Denitrifying Bacteria

The predominant nitrifying bacteria in the hybrid and fixed-film bioreactors were *Nitrosomonas* [ammonia-oxidizing bacteria (AOB)], *Nitrospira* and *Nitrobacter* [nitrite-oxidizing bacteria (NOB)]. The nitrifying bacteria were relatively more abundant in the suspended biomass compared to the fixed-films in the hybrid bioreactor (MBBR SBR). Several phenomena can contribute to the higher relative abundance of the nitrifying bacteria in the suspended solids. These include (a) the oxygen penetration is complete in the flocs to support the growth of nitrifying bacteria; whereas, biofilms have stratified structures with oxic, anoxic and anaerobic zones, (b) the sloughing or erosion of the outer layer of biofilms where nitrifying bacteria are predominant, and (c) the enrichment of nitrifying bacteria found associated with microbial aggregates with improved settleability (low SVI). The conditions in the SBR appeared to enhance the enrichment of fast settling flocs containing nitrifying bacteria. Laureni et al. (2019) have also observed, relatively higher abundance of the nitrifying bacteria in the flocs compared to biofilms of hybrid MBBR SBR.

The *Nitrosomonas* bacteria were relatively more abundant in the fixed-films of the MBBR CSTR compared to the SBR fixed-films. However, the ammonia removal was relatively more stable and elevated in the hybrid bioreactor compared to the MBBR CSTR. The suspended biomass in the hybrid bioreactor complements the nitrification capability of the fixed-film that results in the functional stability of the system. Biofilm sloughing is an integral part of biofilm development that can be triggered depending upon the internal or external stressors (Picioreanu et al., 2001; Aqeel et al., 2019). In conventional fixed-film bioreactors, the biofilm sloughing results in a transient decline in the efficiency of wastewater treatment. Whereas, a major biofilm sloughing can result in biomass washout and system failure.

The relative abundance of NOB is dependent on the AOB in the nitrifying bacterial communities (Winkler et al., 2012). Therefore, the relative abundance of AOB is usually about twofold higher than the NOB (Winkler et al., 2012; Aqeel et al., 2019). In the present study, the relative abundance of AOB was mostly more than 10-fold higher compared to the well-known NOBs (*Nitrobacter* and *Nitrospira*) (Figure 5). However, the abundance of *Nitrospira* (NOB) was relatively higher compared to the *Nitrosomonas* (AOB) in the samples collected during a gradual increase in the influent ammonia concentration. Specifically, the relative abundance of *Nitrospira* was two to threefold higher in the fixed-films of the MBBRs (both SBR and CSTR) on day 120 (Figure 5 and Supplementary Figure S7). Some of the bacterial species related to *Nitrospira* are comammox that are capable of complete ammonia oxidation to nitrate (Daims et al., 2015). Therefore, it is possible that during a gradual increase in ammonia concentration, the comammox *Nitrospira* outcompete *Nitrosomonas* for ammonia oxidation.

Additionally, nitrite loop and ping-pong theories have been proposed to justify the higher abundance of NOB compared to AOB (Winkler et al., 2012; Aqeel et al., 2019). Nitrite loop is described as oxidation of nitrite by NOB to nitrate, followed by a reduction of nitrate by denitrifying bacteria to nitrite. The recycled nitrite is again available for NOB metabolism that results in a higher relative abundance of NOB compared to the AOB. According to the ping-pong theory, the NOB is capable of oxidizing nitrite to nitrate, followed by heterotrophic denitrification of the nitrates. *Rhodanobacter* is capable of complete denitrification that can metabolize nitrite, nitrate and nitrous oxide for complete denitrification (Green et al., 2012; Prakash et al., 2012; Aqeel et al., 2016). In the present study, *Rhodanobacter* was predominant in the BioCord^TM^ SBR and CSTR on days 45 and 300.

The predominant denitrifying bacteria in the hybrid and fixed-film bioreactors were *Rhodanobacter*, *OLB8*, and *Comamonas*. The higher relative abundance of *Rhodanobacter* was also observed in the microbial community of granular sludge in the bioreactors that were seeded with the biomass from the same wastewater treatment plant in an earlier study (Aqeel et al., 2016). This further reinforces that nitrifiers and denitrifiers in biomass at low levels can be easily enriched and selected for in the development of microbial structures and in supporting nitrifying and denitrifying activities.

The abundance of denitrifying bacteria was relatively higher in the fixed-films compared to the suspended biomass of the hybrid bioreactors, and is consistent with the observation that denitrifying bacteria are predominantly present in the anoxic layer of the biofilms (Mahendran et al., 2012; Aqeel et al., 2019). The abundance of the denitrifying bacteria in fixed-films indicates that the sloughing/erosion of the biofilms was resulting in the detachment of bacteria, predominantly from the outer oxic layer of the biofilms. Nitrifying bacteria were predominant in the suspended biomass of the hybrid reactor. The relative distribution of nitrifying and denitrifying bacteria in suspended biomass and fixed-films, respectively, further suggests interactions and possible synergy between the fixed and suspended biomass in biological nutrient removal in hybrid bioreactors (Randall and Sen, 1996; Aqeel et al., 2019).

The relative abundance of the denitrifying bacteria gradually increased in all bioreactors, with increasing influent ammonia concentration and biofilm growth (Figure 5). The anoxic layer of a biofilm increases with the growth of biofilm that can support denitrifying bacteria. However, in the absence of organics in the influent, the observation of dead cells in the fixed-films suggests that the endogenous decay might have supported the growth of the heterotrophic denitrifying bacteria. The development of unfavorable conditions in the deeper layer of the fixed-film limits the growth of aerobic bacteria. Bacteria may produce toxic metabolites that trigger the cell lysis of competitor microbes (Adav et al., 2010; Aqeel et al., 2016). In the present study, bacteria related to *Isosphaeraceae* (phylum, planctomycetes) were predominant in the fixed-film microbial communities. The relative abundance of *Isosphaeraceae* in fixed-film communities increased gradually with biofilm development (Supplementary Figure S5). The bacteria related to *Isosphaeraceae* are capable of synthesizing enzymes that can hydrolyze the complex microorganism-based carbohydrates; and synthesize the secondary metabolites that have potential bacteriocin and antibiotic activity (Kulichevskaya et al., 2016; Ivanova et al., 2017). The resulting soluble organic matter provides a nutrient source for heterotrophic denitrifying bacteria in the anoxic layer. Additionally, experimental and modeling studies indicate that autotrophic NOB produce soluble microbial products (SMP) that may serve as an organic substrate for the growth of heterotrophic denitrifying bacteria (Ni et al., 2011). Heterotrophs are also capable of producing the SMP that can further support the growth of denitrifying bacteria and nitrogen removal (Xie et al., 2012).

There was a gradual increase in nitrate and nitrite accumulation in the bioreactors (Figure 1). This was expected to decline with an anticipated increase in denitrifying bacteria (Figure 5). The nitrate and nitrite removal declined from 80–81% (at start-up) to 2–9% in the SBRs (on day 180 when the NH_4_ in the feed was at 1,000 mg (NH_4_ N/L). The bioreactors were operated under organic carbon limiting conditions (without external organics, but with endogenous decay). Carbon limiting conditions are known to support only partial denitrification (Aqeel et al., 2019) that result in the accumulation of nitrite in the system (Zhang et al., 2020). The accumulation of nitrite during partial denitrification is an exciting avenue for the application of PD/A technology for the treatment of mainstream high strength wastewater (Du et al., 2019; Zhang et al., 2020). The aim of this study was not partial nitritation, or partial denitrification; however, nitrite accumulation was observed in the system that suggests further studies are required to study the system by seeding the fixed-film bioreactors with biomass that have nitrifying and anammox bacteria. Further assessment of the contribution of organic material produced in autotrophic biofilms to the overall treatment process would be valuable. The possibility of sustaining autotrophic nitrogen in high strength waste-streams in the presence of organic substrates warrants further investigation.

## Conclusion

The enrichment of nitrifying bacteria from municipal wastewater biomass was achieved in fixed-film bioreactors, by acclimation of the seed biomass and gradual ramping-up of the influent ammonia concentration. Two types of fixed-film bioreactors (MBBR and BioCord^TM^) in two different configurations (SBR and CSTR) were operated for 306 days. Elevated (up to 99–100%) ammonia removal efficiency was observed at the ammonia loading rate of 2.39 g (NH_4_) N/m^2^.day (750 mg N/L), and was relatively more stable in the MBBR and BioCord^TM^ SBRs compared to the CSTRs. The maximum ammonia loading rate was 3.23 g N/m^2^.day (1,000 mg N/L), where the hybrid MBBR SBR and BioCord^TM^ CSTR removed ammonia at a rate of 3.01 g N/m^2^.day. The nitrifying (*Nitrosomonas*, *Nitrospira*, and *Nitrobacter*) and denitrifying (*Comamonas*, *OLB*8, and *Rhodanobacter*) bacteria were predominant in the microbial communities of the fixed-film and hybrid bioreactors. The presence of dead cells (in the absence of organics in the synthetic feed) suggests endogenous decay supported the growth of the denitrifying bacteria. The limiting organic carbon might have resulted in partial denitrification and accumulation of nitrite in the fixed-film bioreactors. The observation of nitrite accumulation is interesting because it shows that the fixed-film and hybrid bioreactors have the potential to support PD/A processes. Further studies are required to understand the contribution of cellular material (decaying cells, storage polymers, and SMP) in autotrophic ammonia removal from high strength wastewater.

## Data Availability Statement

The datasets generated in this study can be found in online repositories. The names of the repository/repositories and accession number(s) can be found at: https://www.ncbi.nlm.nih.gov/, PRJNA614475.

## Author Contributions

HA conducted the experiments and wrote the first draft. HA and SL contributed to the interpretation of the data, manuscript revision, read, and approved the manuscript. Both authors contributed to the article and approved the submitted version.

## Conflict of Interest

The authors declare that the research was conducted in the absence of any commercial or financial relationships that could be construed as a potential conflict of interest.

## Footnotes

1 https://www.ncbi.nlm.nih.gov/bioproject/PRJNA614475

## Supplementary Material

FIGURE S1 The BioCord^TM^ carrier media, the rings of biocord interwoven on a central cord to support biofilm formation.

FIGURE S2 The attached biomass during the early stage and the relatively mature biofilms on MBBR and BioCord^TM^ carrier media.

FIGURE S3 The sludge volume index (SVI) of the suspended biomass of hybrid bioreactor gradually decreased with increasing influent ammonia concentration.

FIGURE S4 Heat map tree illustrating microbial diversity at **(A)** the bacterial class level, and **(B)** family level.

FIGURE S5 The relative abundance of predominant bacterial families (top 20) in the hybrid and fixed-film bioreactors.

FIGURE S6 The relative abundance of predominant bacterial genera (top 10) in the hybrid and fixed-film bioreactors.

FIGURE S7 The relative abundance and dynamics of predominant **(A)** nitrifying bacteria, and **(B)** denitrifying bacteria with influent ammonia concentration in the hybrid and fixed-film bioreactors.
