# Supplementary material for: Autotrophic Fixed-Film Systems Treating High Strength Ammonia Wastewater
Source: Front Microbiol. 2020 Sep 8;11:551925. doi: 10.3389/fmicb.2020.551925 (PMC7506033; doi:10.3389/fmicb.2020.551925)
Supplement: FIGURE S1 — The BioCordTM carrier media, the rings of biocord interwoven on a central cord to support biofilm formation. [file Data_Sheet_1.PDF]

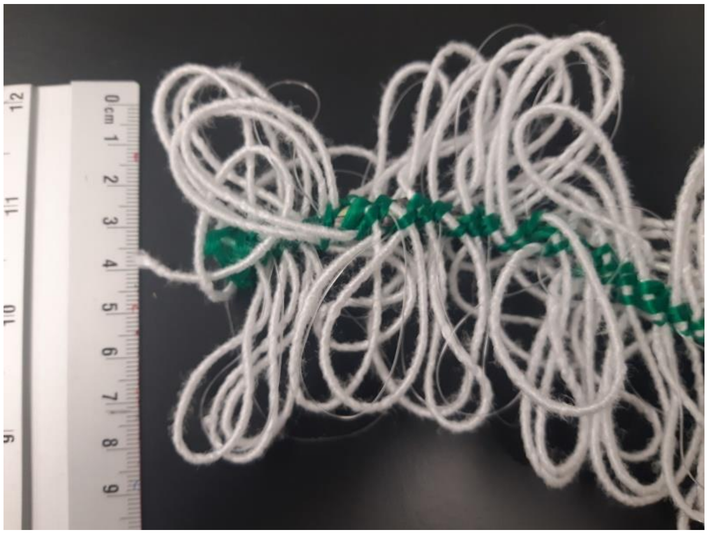

**Fig. S1** The BioCord™ carrier media, the rings of biocord interwoven on a central cord to support biofilm formation.

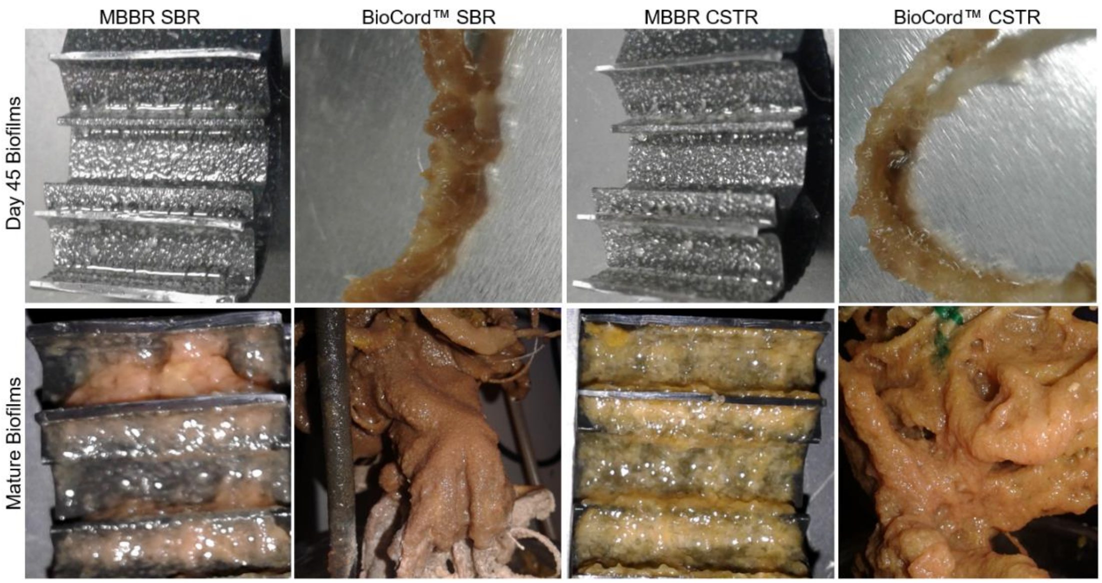


**Fig. S2** The attached biomass during the early stage and the relatively mature biofilms on MBBR and BioCord$^{TM}$ carrier media.

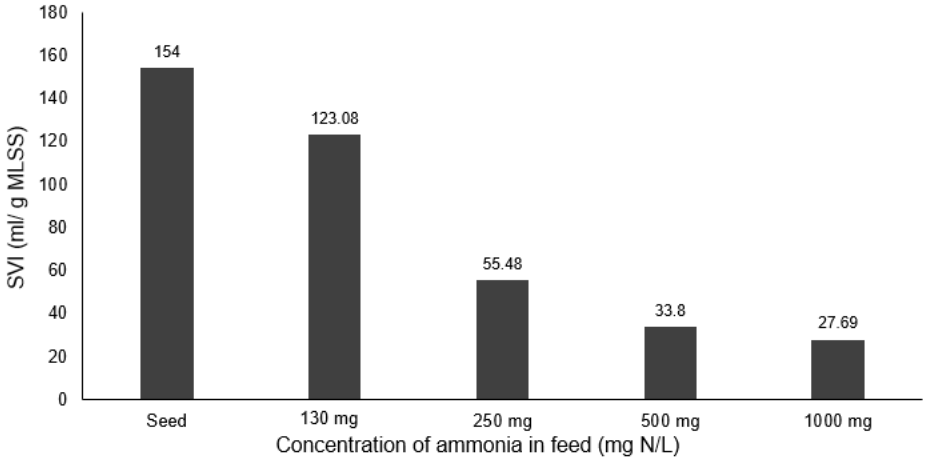


**Fig. S3** The sludge volume index (SVI) of the suspended biomass of hybrid bioreactor gradually decreased with increasing influent ammonia concentration.

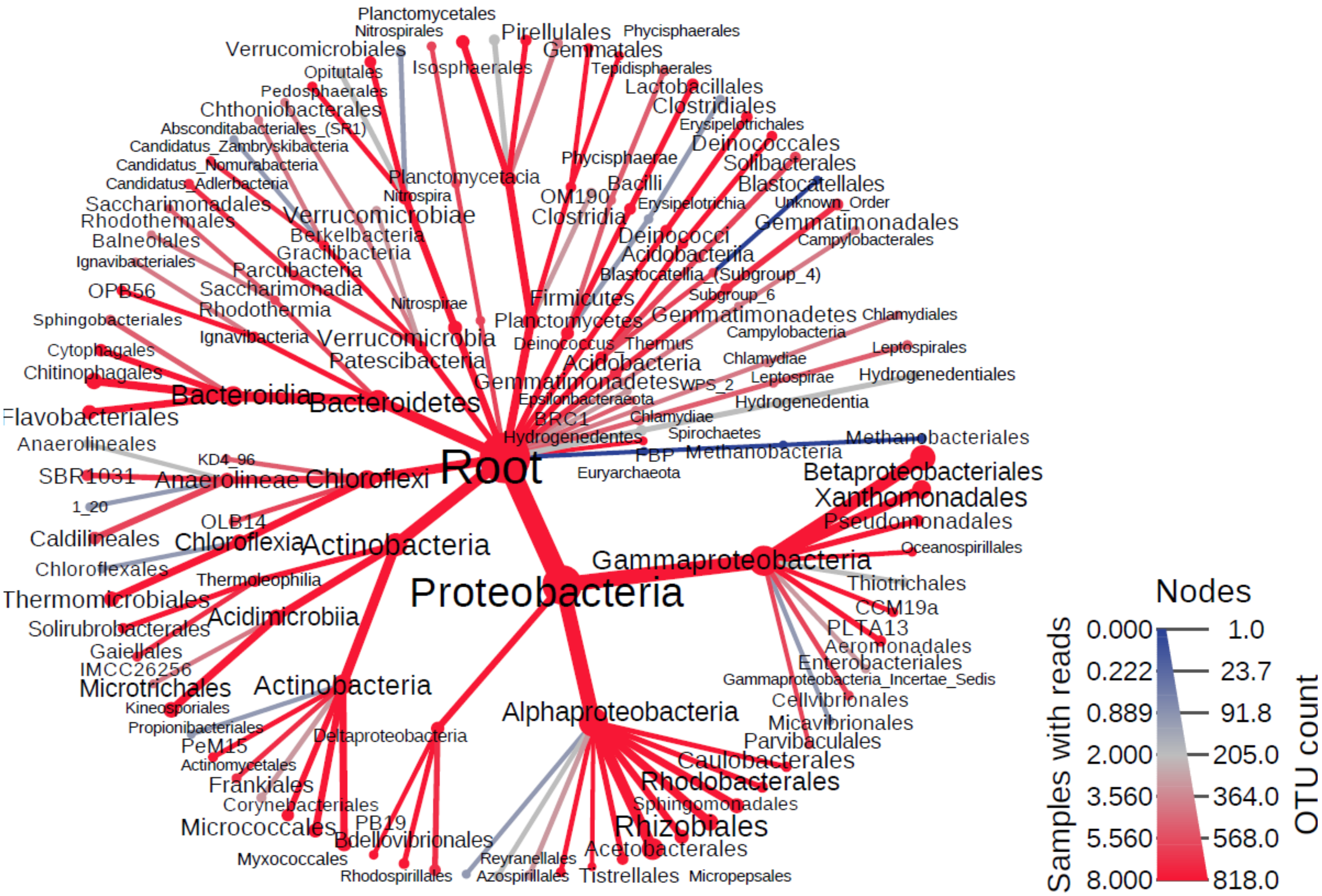
Root
Proteobacteria
Gammaproteobacteria
Alphaproteobacteria
Deltaproteobacteria
Betaproteobacteriales
Xanthomonadales
Pseudomonadales
Oceanospirillales
Thiotrichales
CCM19a
PLTA13
Aeromonadales
Enterobacteriales
Gammaproteobacteria_Incertae_Sedis
Cellvibrionales
Micavibrionales
Parvibaculales
Caulobacterales
Rhodobacterales
Sphingomonadales
Rhizobiales
Acetobacterales
Tistrellales
Micropepsales
Reyranellales
Azospirillales
Rhodospirillales
Bdellovibrionales
PB19
Myxococcales
Actinobacteria
Acidimicrobiia
Thermoleophilia
Microtrichales
IMCC26256
Gaiellales
Solirubrobacterales
Kineosporiales
Propionibacteriales
PeM15
Actinomycetales
Frankiales
Corynebacteriales
Micrococcales
Chloroflexi
Anaerolineae
Chloroflexia
KD4_96
Anaerolineales
SBR1031
1_20
OLB14
Caldilineales
Chloroflexales
Thermomicrobiales
Bacteroidetes
Bacteroidia
Flavobacteriales
Chitinophagales
Cytophagales
Sphingobacteriales
OPB56
Ignavibacteriales
Balneolales
Rhodothermales
Saccharimonadales
Candidatus_Adlerbacteria
Candidatus_Nomurabacteria
Candidatus_Zambryskibacteria
Absconditabacteriales_(SR1)
Chthoniobacterales
Pedosphaerales
Opitutales
Verrucomicrobiales
Nitrospirales
Planctomycetales
Pirellulales
Phycisphaerales
Isosphaerales
Gemmatales
Tepidisphaerales
Lactobacillales
Clostridiales
Erysipelotrichales
Deinococcales
Solibacterales
Blastocatellales
Unknown_Order
Gemmatimonadales
Campylobacterales
Chlamydiales
Leptospirales
Hydrogenedentiales
Methanobacteriales
Planctomycetacia
Nitrospira
Verrucomicrobiae
Berkelbacteria
Gracilibacteria
Parcubacteria
Saccharimonadia
Rhodothermia
Ignavibacteria
Nitrospirae
Verrucomicrobia
Patescibacteria
Phycisphaerae
OM190
Bacilli
Clostridia
Erysipelotrichia
Deinococci
Acidobacteriia
Blastocatellia_(Subgroup_4)
Subgroup_6
Firmicutes
Planctomycetes
Gemmatimonadetes
Campylobacteria
Deinococcus_Thermus
Acidobacteria
Chlamydiae
Leptospirae
Gemmatimonadetes
WPS_2
Epsilonbacteraeota
Hydrogenedentia
BRC1
Chlamydiae
Hydrogenedentes
Spirochaetes
FBP
Methanobacteria
Euryarchaeota
Nodes
Samples with reads
0.000
0.222
0.889
2.000
3.560
5.560
8.000
1.0
23.7
91.8
205.0
364.0
568.0
818.0
OTU count

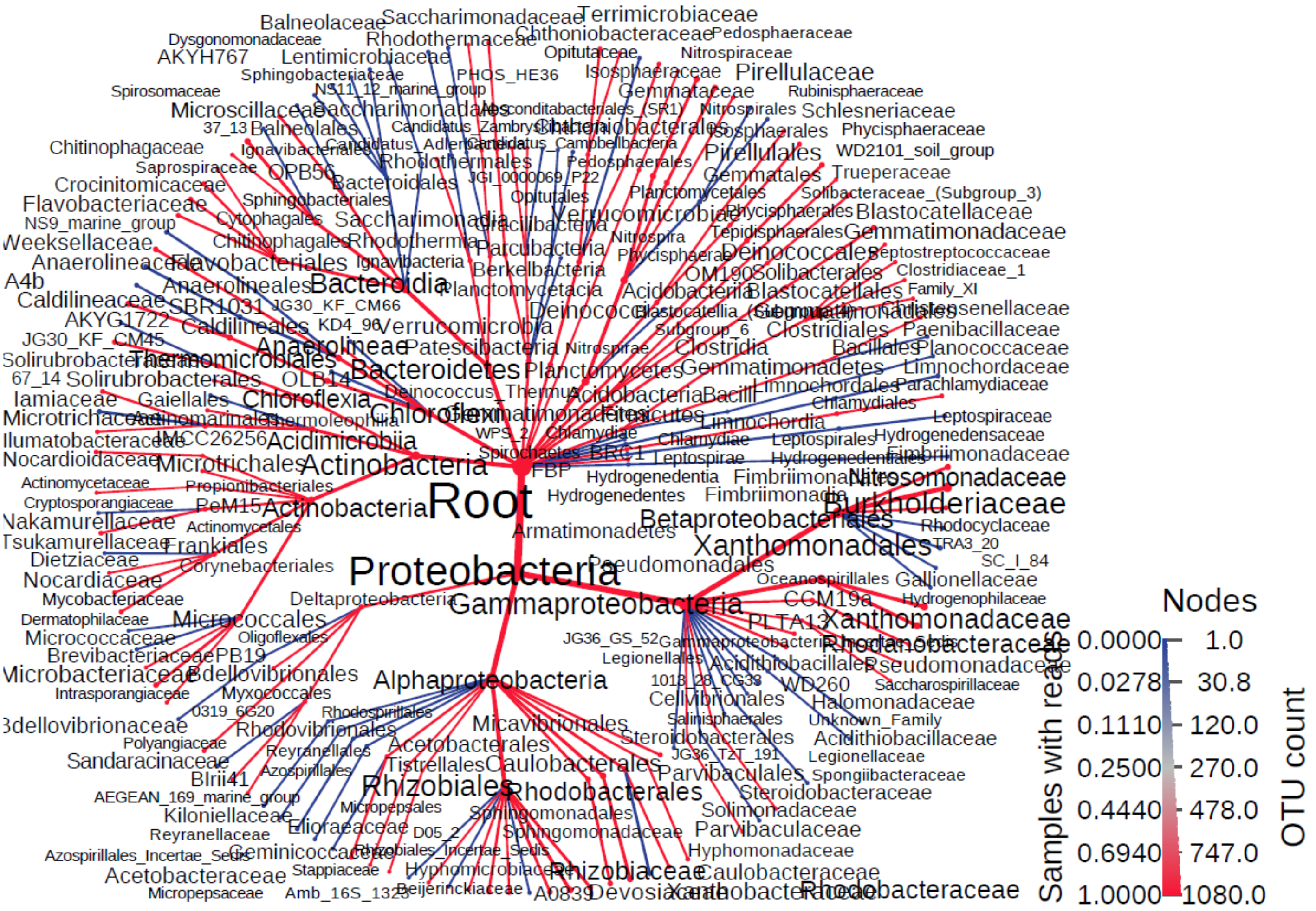


**Fig. S4** Heat map tree illustrating microbial diversity at (a) the bacterial class level, and (b) family level.

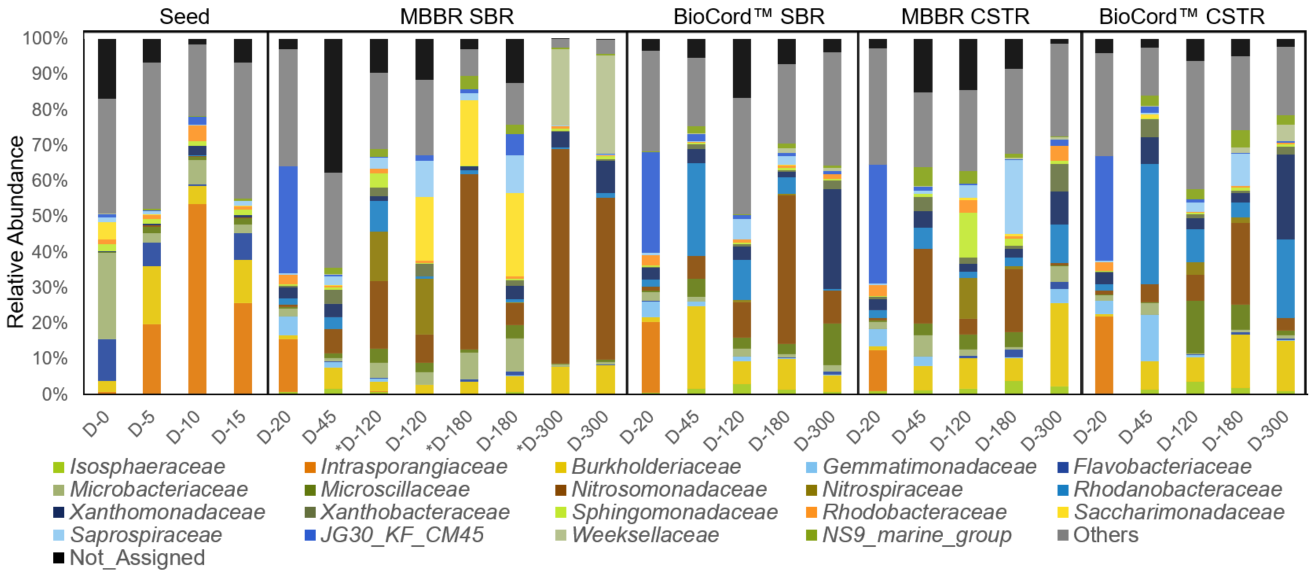


**Fig. S5** The relative abundance of predominant bacterial families (top 20) in the hybrid and fixed-film bioreactors.

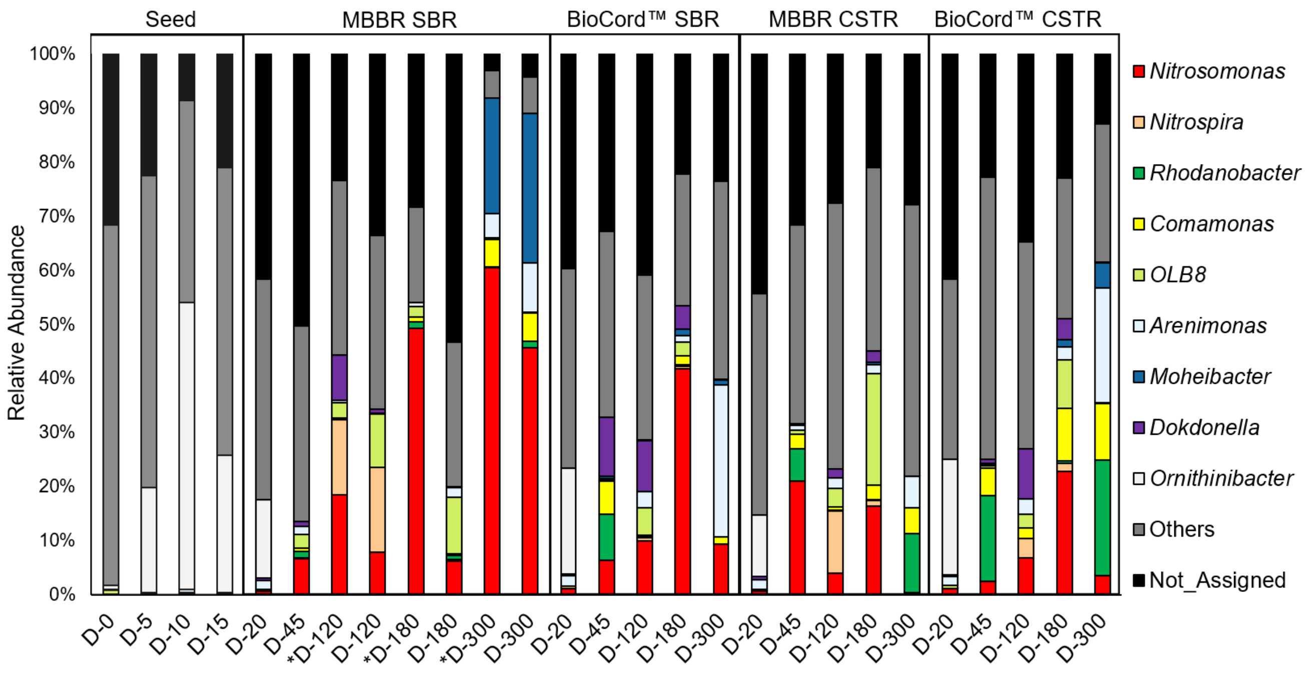


**Fig. S6** The relative abundance of predominant bacterial genera (top 10) in the hybrid and fixed-film bioreactors.

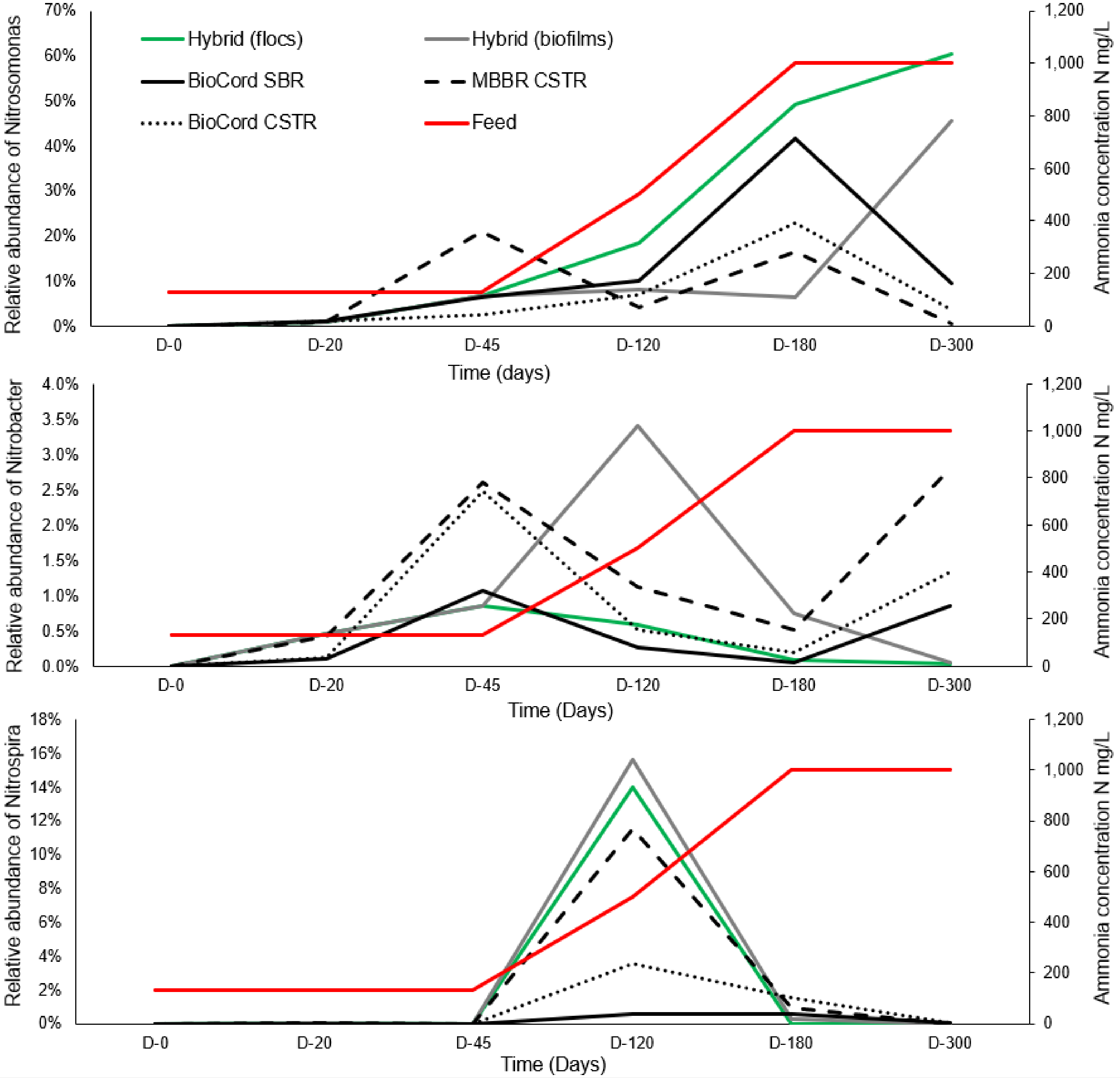

Hybrid (flocs)
Hybrid (biofilms)
BioCord SBR
MBBR CSTR
BioCord CSTR
Feed
Relative abundance of Nitrosomonas
70%
60%
50%
40%
30%
20%
10%
0%
Ammonia concentration N mg/L
1,200
1,000
800
600
400
200
0
D-0
D-20
D-45
D-120
D-180
D-300
Time (days)
Relative abundance of Nitrobacter
4.0%
3.5%
3.0%
2.5%
2.0%
1.5%
1.0%
0.5%
0.0%
Time (Days)
Relative abundance of Nitrospira
18%
16%
14%
12%
10%
8%
6%
4%
2%
0%
Time (Days)

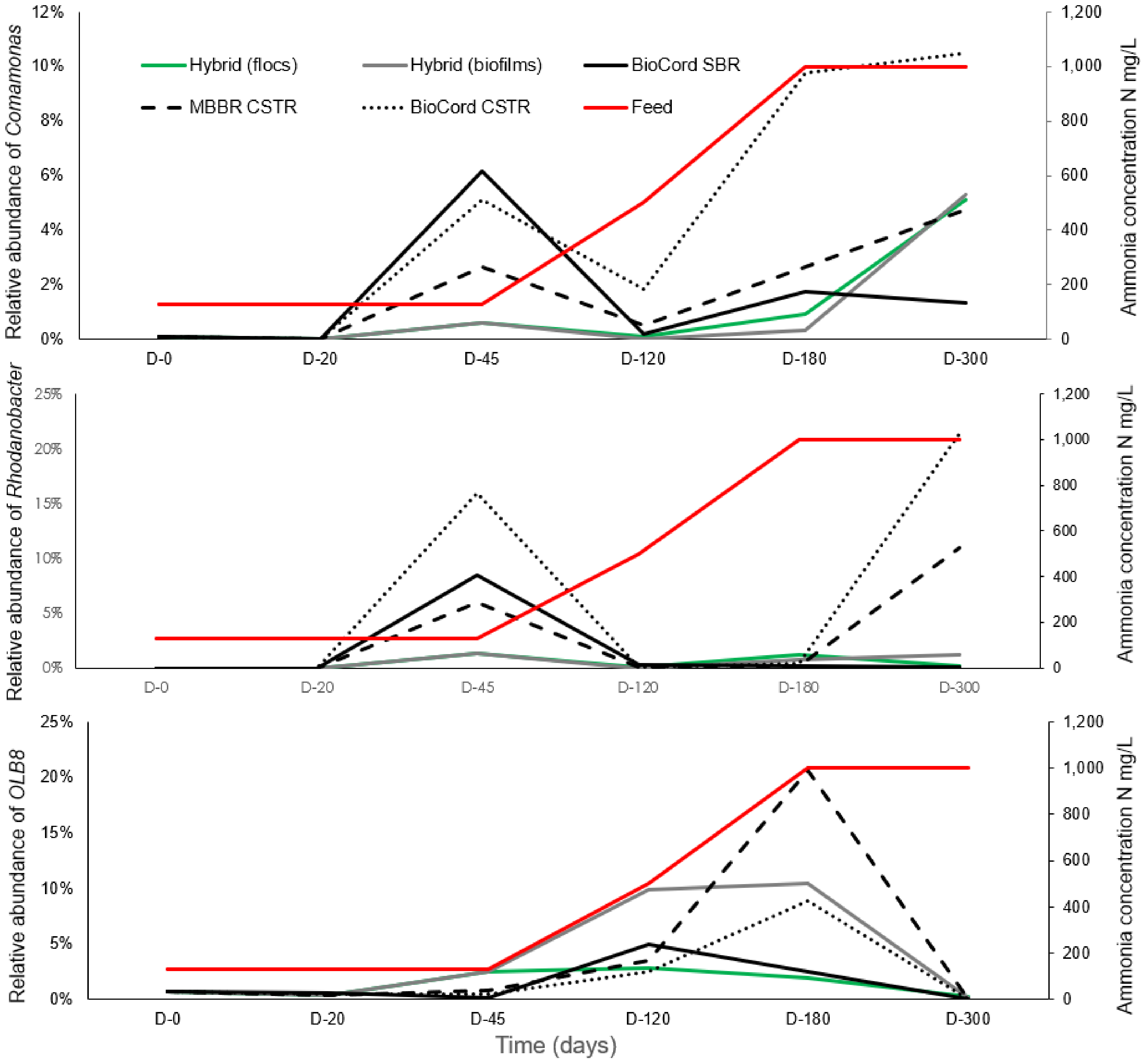


**Fig. S7** The relative abundance and dynamics of predominant (a) nitrifying bacteria, and (b) denitrifying bacteria with influent ammonia concentration in the hybrid and fixed-film bioreactors.
